# Linking growth performance and carcass traits with enterotypes in Muscovy ducks

**DOI:** 10.5713/ab.23.0482

**Published:** 2024-04-25

**Authors:** Qian Fan, Yini Xu, Yingping Xiao, Caimei Yang, Wentao Lyu, Hua Yang

**Affiliations:** 1 College of Animal Sciences & Technology, Zhejiang A & F University, Hangzhou, 311300, China; 2 State Key Laboratory of Hazard Factors and Risk Prevention and Control of Agricultural Product Quality and Safety, Institute of Agro-product Safety and Nutrition, Zhejiang Academy of Agricultural Science, Hangzhou, 310021, China

**Keywords:** Enterotype, Ileal Microbiota, Muscovy Duck, 16S rRNA Gene Sequencing

## Abstract

**Objective:**

Enterotypes (ETs) are the clustering of gut microbial community structures, which could serve as indicators of growth performance and carcass traits. However, ETs have been sparsely investigated in waterfowl. The objective of this study was to identify the ileal ETs and explore the correlation of the ETs with growth performance and carcass traits in Muscovy ducks.

**Methods:**

A total of 200 Muscovy ducks were randomly selected from a population of 5,000 ducks at 70-day old, weighed and slaughtered. The growth performance and carcass traits, including body weight, dressed weight and evidenced weight, dressed percentage, percentage of apparent yield, breast muscle weight, leg muscle weight, percentage of leg muscle and percentage of breast muscle, were determined. The contents of ileum were collected for the isolation of DNA and 16S rRNA gene sequencing. The ETs were identified based on the 16S rRNA gene sequencing data and the correlation of the ETs with growth performance and carcass traits was performed by Spearman correlation analysis.

**Results:**

Three ETs (ET1, ET2, and ET3) were observed in the ileal microbiota of Muscovy ducks with significant differences in number of features and α-diversity among these ETs (p<0.05). *Streptococcus*, *Candida Arthritis*, and *Bacteroidetes* were the presentative genus in ET1 to ET3, respectively. Correlation analysis revealed that *Lactococcus* and *Bradyrhizobium* were significantly correlated with percentage of eviscerated yield and leg muscle weight (p<0.05) while ETs were found to have a close association with percentage of eviscerated yield, leg muscle weight, and percentage of leg muscle in Muscovy ducks. However, the growth performance of ducks with different ETs did not show significant difference (p>0.05). *Lactococcus* were found to be significantly correlated with leg muscle weight, dressed weight, and percentage of eviscerated yield.

**Conclusion:**

Our findings revealed a substantial variation in carcass traits associated with ETs in Muscovy ducks. It is implied that ETs might have the potential to serve as a valuable biomarker for assessing duck carcass traits. It would provide novel insights into the interaction of gut microbiota with growth performance and carcass traits of ducks.

## INTRODUCTION

Farming of ducks and geese has experienced rapid growth due to its cost-effectiveness, short production cycle, and fast rate of development. It has gained significant recognition globally as countries adjust their agricultural industry structures [1]. With the continuous advancement of the economy and improved living standards, there is a rising market demand for duck and goose products. Accordingly, the population of waterfowl reared for commercial purposes is increasing. It is reported that the waterfowl population in China accounts for over 60% of the total population in the world [2]. It is expected that the number of breeding waterfowl will continue to steadily increase in the foreseeable future.

Ducks are an economically important poultry species, therefore, studies of ileal microbes in ducks are attracting more and more attention. The ileum is the main position of digestion, absorption, and nutrient transformation of ducks [3]. The gastrointestinal tract of ducks is inhabited by trillions of commensal bacteria. The supplementation of diets with *Bacillus coagulans* and zinc oxide nanoparticles could improve the gut health, leading to increase the relative weight of leg muscles and influences carcass traits [4], which play a crucial role in facilitating the digestion and absorption of nutrients and energy from diets [5]. Numerous studies have demonstrated the effective role of intestinal bacteria in improving growth performance [6,7]. Our previous research highlighted a significant correlation between gut bacteria and body weight in ducks [3], as well as fat deposition [8]. Administration of *Clostridium butyricum* to newly hatched ducklings was able to modify the intestinal flora, resulting in improved growth performance [9]. To address this issue, the concept of enterotype (ET) was proposed by Arumugam et al [10]. This concept categorizes the intestinal microbial communities of humans into three distinct clusters, referred to as “Enterotypes”, each characterized by a unique assemblage of over-represented bacterial genera. Wu’s study [11] revealed a close relationship between ET and long-term dietary habits, with ET being associated with weight and other phenotypic characteristics in humans. A significant correlation between intestinal flora and carcass traits was found in a study of growing ducks by Li et al [12]. *Clostridium marcescens*, *Clostridium perfringens*, and *Clostridium sporogenes* in the intestine may be involved in changes in liver weight, abdominal fat weight and abdominal fat rate. As a result, the intestinal microflora is thought to have a significant impact on enhancing growth performance.

The composition of the gut microbiota exhibits significant variability among individuals, both over time and in different locations within the gastrointestinal tract, posing a challenge for the practical applications of gut microbiota-based medicine [13]. However, the concept of ET grouping has shown promise in reducing the dimensionality and stratifying gut microorganisms, addressing this challenge to some extent [14]. The notion of ETs has been widely implemented in studies involving various animal species [15–17]. Christensen discovered that *Bacteroides* found within ETs could serve as biomarkers for predicting weight changes in overweight individuals [18]. Additionally, the ileum, positioned as the last part of the small intestine opening into the large intestine at the distal end, plays a pivotal role in enzymatic digestion and absorption of nutrients. It serves as the main site of digestion, absorption, and nutrient transformation in ducks [3]. However, limited research has been conducted on the ETs of ducks. In this study, we identified the ileal ETs of 200 Muscovy ducks based on the 16S rRNA gene sequencing data. The objective of this study was to elucidate the relationship of ETs with growth performance and carcass traits in Muscovy ducks. It would provide novel insights into the interaction of gut microbiota with growth performance and carcass traits of ducks.

## MATERIALS AND METHODS

### Ethics statement

Experimental animal procedures were approved by the Institutional Animal Care and Use Committee of the Zhejiang Academy of Agricultural Sciences (ethics code: ZAAS-2017-009).

### Animals and data collection

A total of 5,000 female newly hatched ducks were raised in cages on plastic nets and fed under standardized conditions. They were fed commercial diets for 70 days as previously described [19]. The composition of the starter and finisher diets used in accordance with previous studies is presented in Table 1 [3,19]. On the 70th day, 200 healthy Muscovy ducks were randomly selected from a population of 5,000 ducks and weighed before euthanization by cervical dislocation following carbon dioxide-induced anesthesia. Three parameters were directly measured, including body weight, dressed weight and eviscerated weight. Subsequently, dressed percentage, percentage of apparent yield, breast muscle weight, leg muscle weight, percentage of leg muscle and percentage of breast muscle were calculated using the method stipulated in Standard NY/T 823-2004 [20], issued by Ministry of Agriculture and Rural Affairs of China. The ileal contents were collected and stored at −80°C.

### DNA extraction and high-throughput sequencing

Genomic DNA from each ileal sample was isolated using the QIAamp DNA Fecal Mini Kit (Qiagen, Valencia, CA, USA) according to the manufacturer’s instructions. The quality and concentration of DNA extracts were assessed through 1% agarose gel electrophoresis and NanoDrop ND-1000 (Thermo Fisher Scientific, Waltham, MA, USA). High-quality DNA was sequenced using next-generation sequencing [21]. Specifically, the V4–V5 region of the bacterial 16S rRNA gene was amplified using barcode fusion forward primer 515F (5′-GTGCCAGCCGGTAA-3′) and reverse primer 907R (5′-CCGTCAATTCMTTRAGTT-3′). The polymerase chain reaction (PCR) conditions were as previously described [3]. Following PCR, amplification products were isolated and identified using a 2% (w/v) agarose gel, and then purified using the GeneJET Gel Extraction Kit (Thermo-Scientific, USA). For sequencing library generation, the Illumina-TruSeq DNA PCR-Free Library Prep Kit (Illumina, San Diego, CA, USA) was employed. The quality of the generated libraries was assessed using an Agilent Bioanalyzer 2100 system and a Quantum 2.0 Fluorometer (Thermo Scientific, USA). Qualified libraries were commercially sequenced using Mingke Biotechnology (Hangzhou, China) on the Illumina NovaSeq platform, yielding 250 bp paired-end reads.

### Data analysis

The sequencing data were analyzed using the QIIME2 microbiome data scientific analysis platform. First, QIIME2 quality filter q-score and deblur noise 16s plugin were employed for raw data quality control, including filtering low quality and noisy sequences, as well as removing chimeras and duplicates.

Based on the obtained effective feature sequences, we utilized QIIME2 along with a plain Bayesian classifier trained on Silva 132 99% operational taxonomic units (OTUs) from the sequence 515F/907R region (https://data.qiime2.org/2018.6/common/silva-132-99-515-806-nb-classifier.qza) for taxonomic classification. To visualize the microbial composition of samples at different levels, the QIIME2 taxonomic unit histogram plugin was used. The Quantitative Insight into Microbial Ecology 2 Taxon Collapse and Feature Table Relative Frequency plugins were employed for calculating the relative abundance of samples at specific taxonomic levels. The ribosomal database project (RDP) classifier was used to annotate the classification information of selected objects [22]. For calculation and visualization of alpha diversity (observed species, Chao 1 estimator, ACE, Shannon, and Simpson indices) GraphPad Prism 8 (GraphPad Software, San Diego, Ca, USA) was used.

### Enterotype clustering

According to the description of Arumugam et al [10], intestinal analysis of the duck involves the assessment of genera abundance in each sample. In short, samples were clustered using the probability distribution distance metric associated with Jensen-Shannon divergence (JSD) and the Division Around Medoids (PAM). The optimal number of clusters was determined utilizing the Calinski–Harabasz (CH) index. The silhouette validation technique was employed to evaluate the stability and robustness of the clusters.

The Chao 1 and Shannon indices were calculated using QIIME2 to describe the alpha diversity of each ET. Statistical analysis was conducted on the dominant microbial taxa at both the phyla and genus levels to illustrate microbial composition in ETs.

### Statistics

Statistical analysis and plotting were performed using SPSS statistical software (version 20.0; International Business Machines Corporation, Armonk, NY, USA) and the GraphPad Prism program (version 6.0; Graphpad Software Inc., USA), respectively.

The data were presented as mean±standard error of the mean. The T-test was used to assess the significance of phenotypes, including body weight, dressed weight, eviscerated weight, dressed percentage, percentage of eviscerated yield, breast muscle weight, leg muscle weight, percentage of leg muscle, percentage of breast muscle among three ETs. For alpha diversity and linear discriminant analysis (LDA), the nonparametric Kruskal-Wallis test was used [23]. Spearman correlation analysis was applied to investigate the correlation among the ileal bacteria and the correlation of differentially abundant bacteria with growth performance and carcass traits [24]. The application of LDA EFfect Size (LEfSe) facilitated the identification of key differentially abundant bacteria. Quality control was carried out based on the original 16S rRNA gene sequencing data, and different sequences are quantified according to taxonomic genes. The Kruskal-Wallis ranking test was used to distinguish the specific differences among ETs. Subsequently, the Wilcoxon rank test was performed among each ET obtained in the previous step to evaluate the consistent differences. Finally, LDA was used to evaluate the effects of key differentially abundant bacteria on different ETs, with the threshold of LDA set at 4.0 [25].

## RESULTS

### Ileum microbial community composition of Muscovy ducks

**Figure 1:**
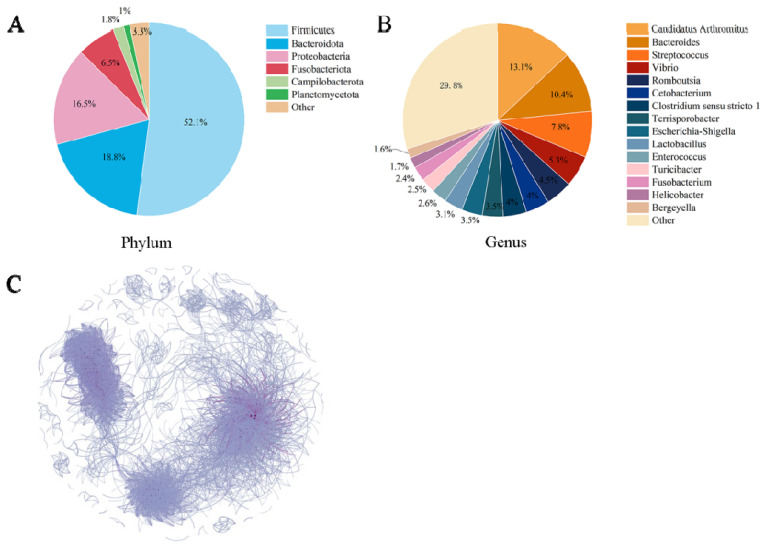
The microbiota structure in the ileum of Muscovy ducks. The top 6 phyla (A) top 15 genera (B) and correlation network of bacteria in the ileum of Muscovy ducks (C). The size of nodes represents relative abundance and the color of connecting lines represents the correlation between bacteria. Spearman’s ρ>0.6; p<0.01.

**Figure 2:**
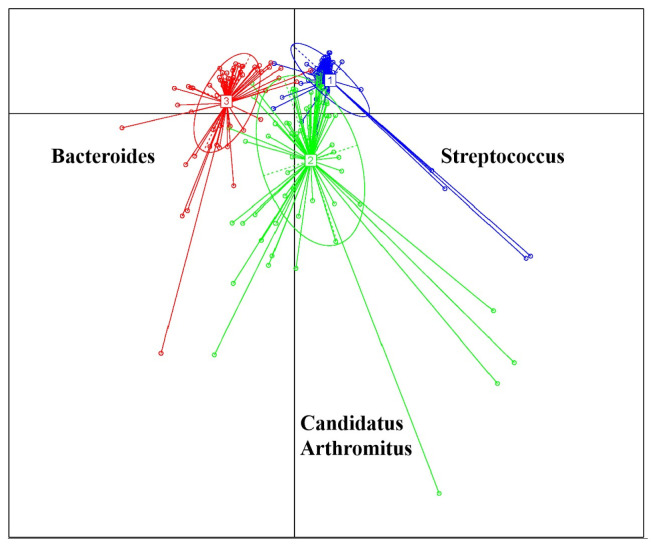
Enterotype clustering in a cohort of 200 Muscovy ducks. The ileum Microbial were clustered into three distinct ETs using the JSD distance metric based on the relative abundances of bacteria at the genus level. ET, enterotype; JSD, Jensen-Shannon divergence.

After quality screening, a total of 8,360,552 DNA sequences were obtained from 200 ducks, with a distribution ranging from 21,243 to 59,481 sequences per sample. These sequences were identified and clustered to 146,731 OTUs at a sequence similarity level of 97%. Employing RDP classifiers for classification analysis, these valid sequences were annotated to 48 phyla and 1,302 genera. Among the samples, the six most abundant phyla, namely Firmicutes, Bacteroidota, Proteobacteria, Fusobacteriota, Campilobacterota, and Planctomycetota, collectively comprised over 95% of the total sequences (Figure 1A). A correlation analysis of the bacteria in the ileum of Muscovy ducks revealed clear clustering into 3 groups (Figure 1C). Therefore, we used the distance measure of JSD and PAM and identified three distinct ETs (Figure 2).

### Enterotypes and their different bacterial community structures

Based on the comparative prevalence of bacteria at the genus level, a total of 200 samples were categorized into three distinct ETs through the utilization of the JSD and PAM distance metric (Figure 2). These formed ETs manifest as clusters, each having a dominant bacterium. Among them, ET1, ET2, and ET3 were characterized by the predominance of *Streptococcus*, *Candidatus Arthromitus* and *Bacteroides*, respectively.

**Figure 3:**
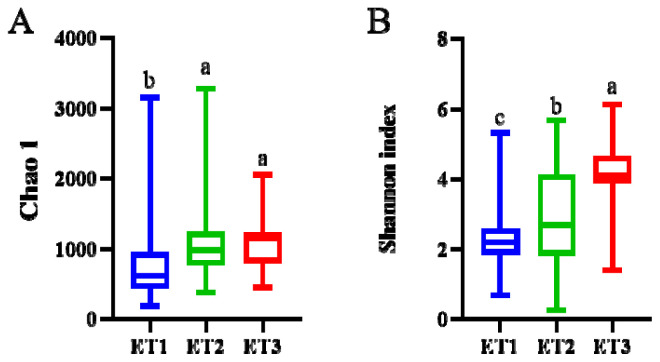
The α-diversity of different ETs. The boxplot shown are means ranges and the first and third quartiles. Different letters indicate a significant difference by nonparametric Kruskal-Wallis test (p<0.05) with the 95% confidence interval. ET, enterotype.

**Figure 4:**
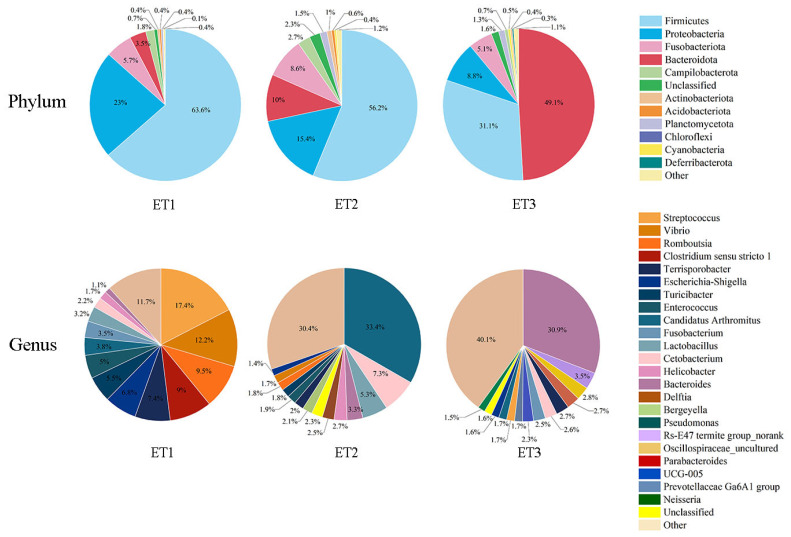
Composition of ET in phylum and genus. This figure mainly shows the major relative abundance in Phylum and genus. ET, enterotype.

Bacterial diversity in the three ETs was estimated by calculating α-diversity (Figure 3). Community richness and microbiota diversity were assessed separately for each ET group using the Chao1 and the Shannon indices. The ET3 had a significantly higher Chao1 and Shannon indices than the other two ETs, indicating that the ET3 had a higher microbial richness and the highest diversity. We analyzed the bacterial distribution of each ETs at the phylum and genus levels. *Streptococcus*, *Candidatus Arthromitus* and *Bacteroides* were the dominant phyla, accounting for about 86% of all bacteria in three ETs, but they were represented in different proportions in each ET (Figure 4; Supplementary Table S1). *Streptococcus* was the most abundant phylum among all three ETs, with the relative abundance in ET1 being even more abundant than that in the other two ETs (p<0.001). The relative abundance of *Candidatus Arthromitus* (p<0.001) and *Bacteroides* (p<0.001) in the ET3 was the highest among all three ETs (Figure 4; Supplementary Table S1).

At the phylum level, the predominant bacterial taxa comprised of Firmicutes, Bacteroidota, Proteobacteria, Fusobacteriota, Campilobacterota, and Patescibacteria. At genus level, the dominant genera observed at the genus level were *Candidatus Arthromitus*, *Bacteroides*, *Streptococcus*, *Vibrio*, *Romboutsia*, *Cetobacterium*, *Clostridium sensu stricto 1*, *Terrisporobacter*, *Escherichia-Shigella*, *Lactobacillus*, *Enterococcus*, and *Turicibacter*. Remarkable discrepancies in all of the dominant bacteria and their relative abundances among the ETs were observed (Figure 4). In ET1, *Streptococcus* and *Vibrio* emerged as the dominant genera, constituting 17.40% and 12.20% of the total bacterial population, respectively. The ET2 exhibited *Candidatus Arthromitus* as the prevailing genus, comprising 33.39% of the total bacterial population. Conversely, *Bacteroides* emerged as the dominant genus in ET3, accounting for 30.94% of the total bacterial population (Figure 4). Notably, the dominant bacteria observed within each ET were consistent with the main bacteria identified in their respective groups. Specifically, *Streptococcus*, *Candidatus Arthromitus*, and *Bacteroides* emerged as the dominant bacteria in ET1, ET2, and ET3, respectively, corroborating the findings presented in Figure 2.

**Figure 5:**
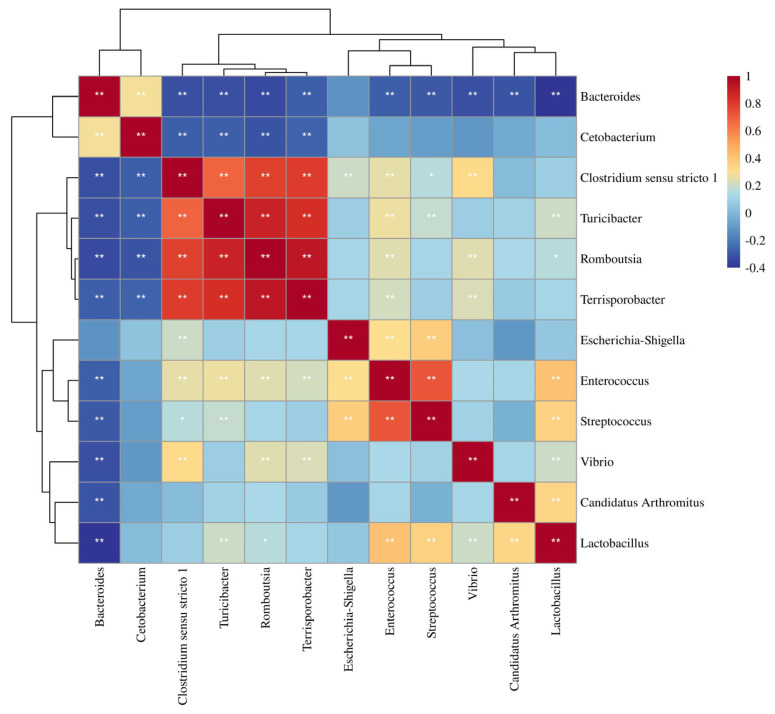
Correlation matrix showing the Spearman’s rank correlations among the most abundant genera. Spearman’s rank correlation coefficients (ρ) range from −0.4 to 1 corresponding to a strongly positive to a strongly negative correlation respectively.

Correlations among the major genera in relative abundance were determined based on Spearman rank correlation (Figure 5). A strong positive correlation was observed between *Clostridium sensu stricto 1*, *Turicibacter*, *Romboutsia*, and Terrisporobacter (Spearman rank correlation coefficients (ρ) were 0.70, 0.75, and 0.89, respectively), while *Cetobacterium* and *Bacteroides* were negatively correlated with almost all other genera (ρ ranged from −0.07 to −0.52).

### Correlation of enterotypes with growth performance and carcass traits

**Figure 6:**
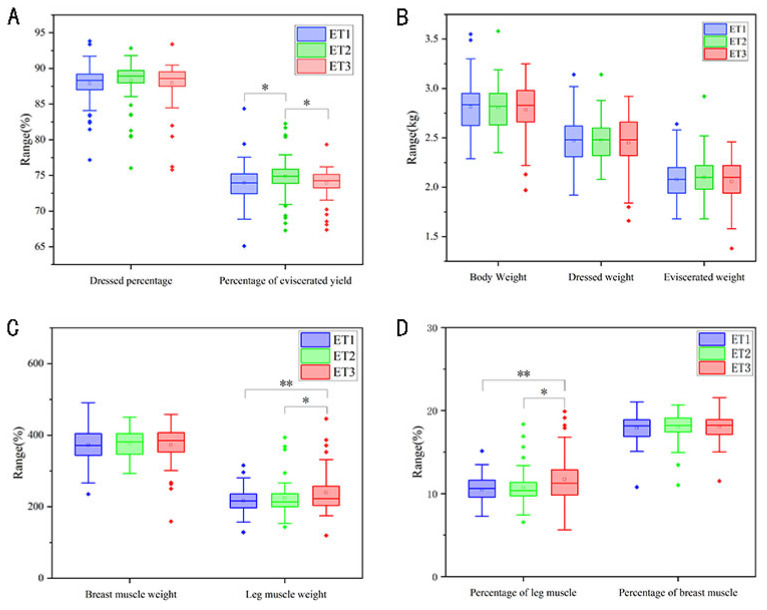
Growth performance of ducks in different ETs. ET, enterotype. * p<0.05; ** p<0.01.

By collating and comparing the growth performance and carcass traits of the three ETs in 200 Muscovy ducks, we found significant differences in the percentage of eviscerated yield, leg muscle weight, and percentage of leg muscle had among the three ETs. However, there was no significant differences in body weight and dressed weight among the three ETs. Specifically, ET3 exhibited significantly higher leg muscle weight and percentage of leg muscle compared to the other two ETs (Figure 6).

**Figure 7:**
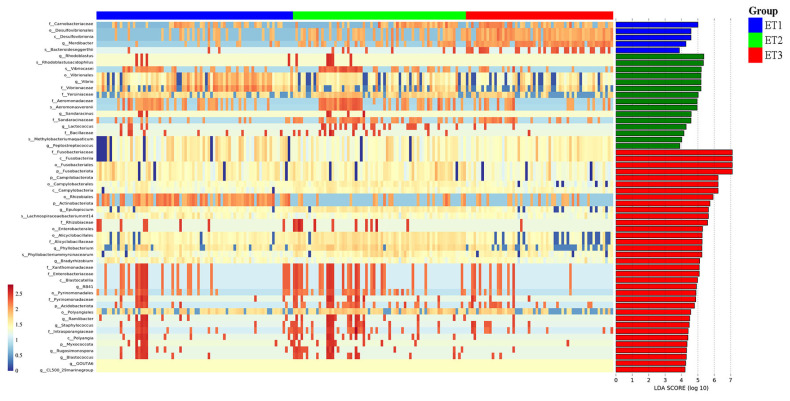
Differentially abundant bacteria in three ETs of Muscovy ducks. Lefse analysis was performed using p<0.05 and a LDA score of 4.0 as the threshold. ET enterotype; LDA, linear discriminant analysis.

To explore the correlation of ETs with growth performance and carcass traits, we identified 16 differentially enriched bacterial genera with LDA>4.0 (Figure 7).

**Figure 8:**
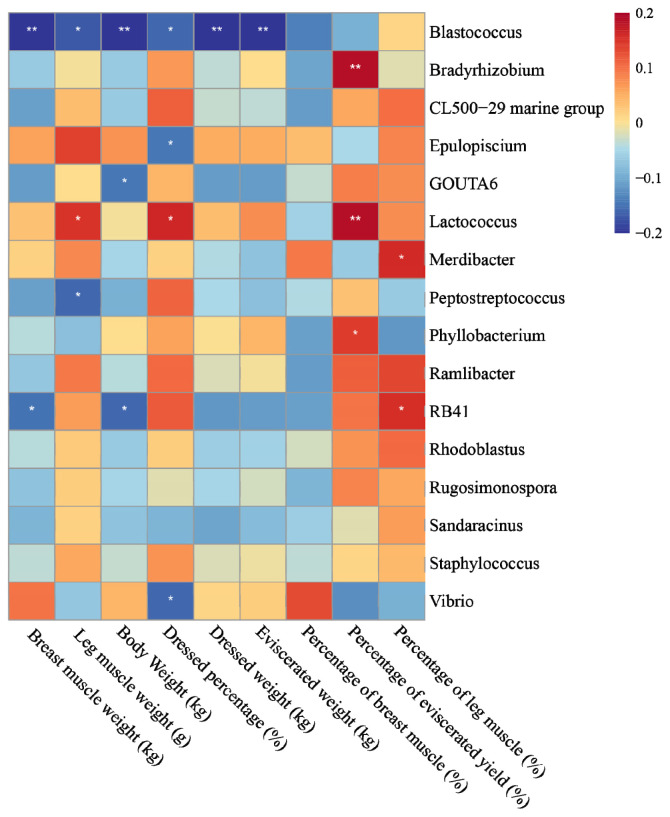
Correlation matrix showing Spearman’s rank correlation between the most abundant genera and phenotype. Spearman’s rank correlation coefficients (ρ) range from −0.2 to 0.2 corresponding to a strongly positive to a strongly negative correlation respectively.

Further insights were gained by examining the association of the distinct bacterial taxa with growth performance and carcass traits among the three ETs. Our analysis unveiled significant negative correlations of *Blastococcus* with leg muscle weight, body weight, dressed weight, dressed percentage, and eviscerated weight, along with a positive correlation of *Lactococcus* with leg muscle weight, dressed weight, and percentage of eviscerated yield. Additionally, *Epulopiscium* and *Vibrio* displayed negative correlations with dressed percentage. Notably, the relationship between *Bradyrhizobium* and percentage of eviscerated yield exhibited an extremely significant and positive correlation. Encouragingly, the relative abundance distribution of these genera across the three ETs aligned with our findings regarding the intestinal-phenotype relationship (Figure 8).

## DISCUSSION

With the development of effective analytical methods, 16S rRNA gene sequencing techniques could provide insights into the complex biological functions of the microbiota within the intestinal niche [26]. In the present study, we employed 16S rRNA gene sequencing to analyze the intestinal contents in the ileum of Muscovy ducks. Previous studies have consistently identified *Firmicutes* and *Bacteroides* as the two most abundant phyla in the intestine of Muscovy ducks [27–29]. At class level, *Clostridia* and *Bacteroidia* are reported to be dominant in the ileum of Muscovy ducks [30], which is consistent with our research. In the present study, we delved into the relative abundance of key bacterial genera within the ileal microbial community of Muscovy ducks. The concept of ET was first defined as “densely populated areas in a multidimensional space of community composition” by Arumugam et al [10], and 3 ETs were identified in the human gut microbial community. It has been reported that different ETs are not influenced by geographical location, sex, or age but are driven by the relative abundance of dominant bacteria genera [31]. Although the 200 ducks were raised under the same breeding condition and management, variation in body weights were observed. Similarly, the bacterial composition in the ileum of Muscovy ducks showed differences among 200 ducks, leading to the identification of 3 ETs in the present study. *Streptococcus*, *Candidatus Arthromitus*, and *Bacteroides* emerged as the presentative genera of ET1 (n = 76), ET2 (n = 67), and ET3 (n = 57), respectively. Notably, we observed significant differences among these ETs in percentage of eviscerated yield, leg muscle weight, and percentage of leg muscle. These differences can potentially be attributed to variations in the relative abundance of the genera present within each ET.

The concept of ETs, initially proposed to categorize the human gut microbiota, has provided valuable insights into understanding and manipulating complex gut microbial communities [10]. This concept has been extended to encompass other animal species. Since the introduction of the ET concept, it has been increasingly utilized in studying intestinal bacteria in various animal species. However, most of these studies have focused on mammals like pigs [32] and chimpanzees [15], with limited applications in animals such as poultry. In the case of chimpanzees, their microflora was classified into three distinct clusters, referred to as ETs, based on genus-level composition [15]. The key bacterial groups contributing to each cluster were *Faecalibacterium* in chimpanzee ET1, *Lachnospiraceae* in ET2, and *Bulleidia* in ET3, exhibiting similarities to human ETs. In a study conducted on Jinhua pigs, three ETs were identified [33]. The primary genera among ET1, ET2, and ET3 were *Lactobacillus*, *Clostridium sensu stricto 1*, and *Bacteroides*, respectively. In the duodenum of broiler chickens, *Proteobacteria*, *Firmicutes*, and *Actinomycetes* were dominant in the ET1 and ET2 groups, while *Firmicutes* and *Verrucomicrobia* were more abundant in the ET3 group. *Bacteroides* was the main microorganism genus overrepresented in group ET1 broilers. *Escherichia-Shigella* was identified as another driving genus in the ET1 group, and the ET2 group was overrepresented by *Ochrobactrum* and *Rhodococcus*. The proportion of *Bacillus* and *Akkermansia* in broiler ET3 group was notably high, with the proportion of *Akkermansia* in human ET3 also being elevated [31]. In our study, the bacteria in the ileum of Muscovy ducks were divided into three ETs, which were *Streptococcus*, *Candidatus Arthromitus*, and *Bacteroides*. Enterotype classification might be species-specific. The divergence from the findings reported in the aforementioned studies may be attributed to species differences among hosts, along with potential nonsignificant distinctions among closely related species, as observed in the case of chimpanzees and humans.

Different ETs might show different growth performance and carcass traits as previously described [34]. As expected, our experimental results indicated that different ETs showed obvious differences in carcass traits (p<0.05), particularly in leg muscle weight, percentage of leg muscle, and percentage of eviscerated yield (Figure 6). We suspect that these differences may be attributed to the variation in intestinal microflora across ETs. Long-term eating habits could lead to differences in the clustering of ETs, and with age, ETs may also change. Different ETs will have an impact on the health of bees, including pathogen defense and nutrition [35]. Soo In Choi found that plateau pikas with different ETs have different heat-producing abilities to resist cold environment, and *Lachnospiraceae*, the main genus in ET2, was associated with larger body weight in cold areas [36]. Similar findings have been reported in other studies, indicating ETs might have the potential to predict carcass traits, which could be beneficial for the grading of duck meat. Lu discovered that the intestinal flora in pigs can be categorized into two ETs, which might be associated with backfat thickness and daily weight gain [37]. Wang observed that certain strains of *Prevotella* present in the intestine of pigs played a direct role in feed conversion rate and contributed to the host’s nutrition supply. *Streptococcus* and *Lactobacillus* were found to be associated with the growth performance of animals, promoting animal growth when colonized the intestine [38]. This aligns with our comparison of body weight among ducks with three different ETs, where the body weight of ET1, dominated by *Streptococcus*, was somewhat higher than that of the other two groups. *Prevotella* members, typically associated with plant-based diets and fiber digestion, may play a potential role in carbohydrate degradation and fat regulation [11]. Research has shown that microbial community structures, which contribute to functional and ecological characteristics, vary among ETs [39]. In the chicken duodenum, dominant bacteria in ET2, such as *Haematitum* and *Staphylococcus aureus*, might work together to degrade lignocellulosic biomass, producing monosaccharides or short-chain fatty acids. This process could facilitate nutrient absorption by the host and promote adipose tissue synthesis [30]. The dominant bacteria within ETs have a close association with the expression of poultry phenotypes. Evidence presented by Danzeisen et al [40] suggests that the interaction between *Candidatus Arthromitus* and epithelial cells may contribute to the early health of the digestive system. *Bacteroides* played an important role in metabolizing polysaccharides and oligosaccharides to provide nutrients and vitamins for the host and other intestinal microbial populations [41,42]. We believe it is possible to grade duck meat using ET as a biomarker for carcass traits.

Comparing various strains of three different ETs, we have identified certain strains that have been under-investigated in poultry intestines, such as *Peptostreptococcus*, *Blastococcus*, and *Epulopiscium*, among others. *Peptostreptococcus* is commonly found in the digestive tracts of ruminants and possesses the ability to metabolize tryptophan in the rumen, leading to the production of indole and its derivatives [43]. Certain indole compounds play a role in enhancing intestinal barrier function, boosting immune response, and exerting anti-inflammatory effects to improve metabolism [44]. *Blastococcus* interacts closely with *Aeromonas* and *Mannheimia*, contributing to the regulation of permeability [45]. *Epulopiscium* thrive in the intestines of herbivorous monitor lizards and ants, possibly aiding in the digestion of plant fibers [46]. These bacteria possess a reported diurnal life cycle, closely connected to the daily activities of their host organisms [47]. We assume ETs may affect carcass traits due to differential abundant bacteria.

Upon conducting correlation analysis between different bacterial strains within the three ETs and their phenotypic traits, we discovered significant associations between these bacteria and the phenotypic characteristics of Muscovy ducks. *Lactococcus* showed a positive correlation with leg muscle weight, dressed percentage, and the percentage of eviscerated yield. Certain *Lactococcus* strains have the capacity to modulate adipose tissue metabolism and counteract diet-induced obesity [48]. ET3 exhibited the highest content of *Lactococcus*, resulting in both the highest leg muscle weight and dressed percentage among the three groups. The lower percentage of eviscerated yield in ET3, compared to ET2, may be attributed to the distinctively positive relationship between *Bradyrhizobium* and percentage of eviscerated yield. Furthermore, the content of *Lactococcus* in ET2 is significantly higher than that in ET3. However, relevant information regarding the role of *Bradyrhizobium* in the intestine is scant. The three ETs exhibited varying microbial community structures, which subsequently influenced population-level functions in the intestine. This interplay may contribute to differences in the carcass traits of Muscovy ducks.

## CONCLUSION

Collectively, we stratified the ileal bacteria of Muscovy ducks and identified three ETs characterized by dominant genera *Streptococcus*, *Candidatus Arthromitus*, and *Bacteroides*. Notably, our findings indicated a significant variability between the ET of ducks and their carcass traits. Carcass traits of ducks result from complex interactions of intestinal flora, and that ETs may serve as a biomarker for carcass traits of ducks. These findings would provide a new insight into the interaction of gut microbiota with growth performance and carcass traits of ducks.

## Figures and Tables

**Table 1:** Ingredients and nutrient levels of diets

Items	Starter	Finisher
Ingredients (%)		
Corn	58.90	56.50
Soybean meal	28.00	20.00
Wheat	7.27	18.00
Soybean oil	2.05	1.85
Sodium carbonate	1.14	1.16
Dicalcium phosphate	0.68	0.64
Lysine	0.285	0.315
Methionine	0.265	0.235
NaCl	0.40	0.24
Choline chloride	0.06	0.06
Vitamin and trace mineral premix^1)^	1.00	1.00
Calculated nutrients levels (%)		
Metabolizable energy (MJ/kg)	12.12	11.58
Crude protein	20.50	16.50
Calcium	0.86	0.95
Phosphorus	0.53	0.52
Lysine	0.89	0.92
Methionine	0.51	0.49

1) The premix provided per kilogram of total diet: vitamin A 10,000 IU; vitamin D_3_ 2,100 IU; vitamin E 15 IU; vitamin K_3_ 1 mg; vitamin B_1_ 2 mg; vitamin B_2_ 4 mg; vitamin B_6_ 3 mg; vitamin B_12_ 0.005 mg; nicotinic acid 40 mg; pantothenic acid 10 mg; folic acid l mg; biotin 0.3 mg; choline 2,000 mg; Fe 120 mg; Cu 5 mg; Mn 60 mg; Zn 25 g; I 0.3 mg; Se 0.2 mg.

## Data Availability

The original sequencing reads data are available in the NCBI database under accession number: PRJNA762153. The data of growth performance and carcass traits generated and/or analyzed during the study can be obtained from the corresponding authors according to reasonable requirements.

## SUPPLEMENTARY MATERIAL

**Supplementary Table S1**. Differentially abundant bacterial phyla in three enterotypes
