# Supplementary material for: Linking growth performance and carcass traits with enterotypes in Muscovy ducks
Source: Anim Biosci. 2024 Apr 25;37(7):1213–24. doi: 10.5713/ab.23.0482 (PMC11222842; doi:10.5713/ab.23.0482)
Supplement: Supplementary file 1 [file ab-23-0482-Supplementary-Table-1.pdf]

Supplementary Material

1 Supplementary Tables

Table S1. Differentially abundant bacterial phyla in three enterotypes

Phylum	ET1	ET2	ET3	SEM	P-Value
Firmicutes（%）	63.5455a	56.1274a	31.0974b	0.1700	<0.0001
Bacteroidota（%）	3.4730c	10.0179b	49.0551a	0.2464	<0.0001
Proteobacteria（%）	22.9525a	15.4157b	8.8490c	0.0706	<0.0001
Fusobacteriota（%）	5.7063a	8.5873a	5.0893a	0.0187	0.3859
Campilobacterota（%）	1.7793a	2.7500a	0.6862a	0.0103	0.3549
Patescibacteria（%）	0.3873a	0.0202b	0.0139c	0.0021	<0.0001

[a, b, c] The different superscript letters in the same row represent a significant difference ($P < 0.05$). Abbreviation: ET, enterotype.
